# Improvement of Sidestream Dark Field Imaging with an Image Acquisition Stabilizer

**DOI:** 10.1186/1471-2342-10-15

**Published:** 2010-07-13

**Authors:** Gianmarco M Balestra, Rick Bezemer, E Christiaan Boerma, Ze-Yie Yong, Krishan D Sjauw, Annemarie E Engstrom, Matty Koopmans, Can Ince

**Affiliations:** 1 Department of Translational Physiology, Academic Medical Center, University of Amsterdam, Meibergdreef 9, 1105 AZ Amsterdam, The Netherlands; 2 Department of Internal Medicine and Medical Intensive Care, University Hospital Basel, Petersgraben 4, 4031 Basel, Switzerland; 3 Department of Intensive Care, Erasmus Medical Center, 's-Gravendijkwal 230, 3015 CE Rotterdam, The Netherlands; 4 Department of Intensive Care, Medical Center Leeuwarden, Postbus 888, 8901 BR Leeuwarden, The Netherlands; 5 Department of Cardiology, Academic Medical Center, University of Amsterdam, Meibergdreef 9, 1105 AZ Amsterdam, The Netherlands

## Abstract

**Background:**

In the present study we developed, evaluated in volunteers, and clinically validated an image acquisition stabilizer (IAS) for Sidestream Dark Field (SDF) imaging.

**Methods:**

The IAS is a stainless steel sterilizable ring which fits around the SDF probe tip. The IAS creates adhesion to the imaged tissue by application of negative pressure. The effects of the IAS on the sublingual microcirculatory flow velocities, the force required to induce pressure artifacts (PA), the time to acquire a stable image, and the duration of stable imaging were assessed in healthy volunteers. To demonstrate the clinical applicability of the SDF setup in combination with the IAS, simultaneous bilateral sublingual imaging of the microcirculation were performed during a lung recruitment maneuver (LRM) in mechanically ventilated critically ill patients. One SDF device was operated handheld; the second was fitted with the IAS and held in position by a mechanic arm. Lateral drift, number of losses of image stability and duration of stable imaging of the two methods were compared.

**Results:**

Five healthy volunteers were studied. The IAS did not affect microcirculatory flow velocities. A significantly greater force had to applied onto the tissue to induced PA with compared to without IAS (0.25 ± 0.15 N without vs. 0.62 ± 0.05 N with the IAS, p < 0.001). The IAS ensured an increased duration of a stable image sequence (8 ± 2 s without vs. 42 ± 8 s with the IAS, p < 0.001). The time required to obtain a stable image sequence was similar with and without the IAS. In eight mechanically ventilated patients undergoing a LRM the use of the IAS resulted in a significantly reduced image drifting and enabled the acquisition of significantly longer stable image sequences (24 ± 5 s without vs. 67 ± 14 s with the IAS, p = 0.006).

**Conclusions:**

The present study has validated the use of an IAS for improvement of SDF imaging by demonstrating that the IAS did not affect microcirculatory perfusion in the microscopic field of view. The IAS improved both axial and lateral SDF image stability and thereby increased the critical force required to induce pressure artifacts. The IAS ensured a significantly increased duration of maintaining a stable image sequence.

## Background

Orthogonal Polarization Spectral (OPS) imaging and its successor Sidestream Dark Field (SDF) imaging are optical techniques allowing microscopic assessment of microcirculatory density and perfusion in clinical settings [1,2]. These non-invasive intravital imaging modalities have been used in studies for monitoring the severity of shock and efficacy of resuscitation in various patient groups [3-6]. However, as both OPS and SDF imaging technologies are incorporated into hand-held microscopes some operational issues arise in terms of axial and lateral instability of the microscope probes, potentially causing pressure artifacts and image drifting, respectively.

Reductions in sublingual microcirculatory density and perfusion have been associated with patient morbidity and mortality [6]. Correcting these microcirculatory parameters has become the focus of new clinical studies aiming at resuscitating the microcirculation rather than the macrocirculation, using vasoactive agents such as nitroglycerin [7,8]. Hence, microcirculatory images are gaining a more prominent role in clinical monitoring and their accurate interpretation is essential and relies heavily on the quality of the images [9,10]. In this light, the current microcirculatory image acquisition guidelines dictate a minimal recording time of 20 s to allow adequate analysis of microcirculatory density and perfusion [11]. Image drifting, due to the difficulty in holding the tip of the device in one place however, makes this particularly difficult both in sedated and in awake patients. Furthermore, pressure artifacts caused by the physical contact and pressure of the microscope probe to the mucosal tissue can alter mucosal capillary blood flow thereby limit the use of the captured images for determination of microcirculatory perfusion.

Lindert et al. addressed these technical issues associated with hand-held microscopy before by developing an image acquisition stabilizer (IAS) for the OPS imaging device [12]. Their IAS consisted of a ring placed around the tip of the OPS probe through which negative pressure was applied securing the IAS onto the mucosal tissue. The negative pressure appeared not to influence flow patterns of the microcirculation within the microscopic field. However, whether the IAS minimized image drift or induction of pressure artifacts was not evaluated. In addition their IAS was not validated in terms of clinical applicability and utility, including the ease with which the device could be sterilized and cleaned for multiple uses as well as fitting piping of vacuum sources available at the bed-side.

In the present study we developed, evaluated, and validated an IAS for the SDF device. In a study by Goedhart et al., the SDF imaging device was shown to provide microcirculatory images of superior quality with respect to the OPS device [13]. In combination with an IAS, this microcirculatory imaging setup should provide high quality microcirculatory images of sufficient duration and stability. The IAS was designed and fabricated to adhere to clinical requirements. The application of the IAS was validated by measuring 1) the effects of application of peripheral negative pressure on microcirculatory perfusion, 2) the force required for induction of pressure artifacts with and without the IAS, 3) the time required to attain a stable image, and 4) the time that a stable image could be maintained. Then, to demonstrate the clinical applicability of the SDF setup with the IAS, simultaneous bilateral sublingual SDF measurements were conducted in critically ill patients undergoing a standard lung recruitment maneuver with one hand-held SDF device and one SDF device mounted in a mechanical arm and equipped with the IAS whereby stability of acquired images was evaluated.

## Methods

The study protocol was approved by the local medical ethics committee of the Medical Center of Leeuwarden. Written informed consent was obtained from all studied subjects respectively their closest relatives. The study was done in compliance with the principles established in the Helsinki Declaration.

### Sidestream dark field image acquisition and analysis

Sublingual microcirculatory density and perfusion were monitored using an SDF imaging device (Microvision Medical BV, Amsterdam, the Netherlands). A detailed description of the SDF technology is provided elsewhere [13]. Briefly, in SDF imaging, the tissue is illuminated with green light emitting diodes (LEDs) concentrically placed around the central microscopy objective to provide SDF illumination. The lens system in the core of the objective is optically isolated from the illuminating outer ring thus preventing the microcirculatory image from contamination by tissue surface reflections. To further improve the imaging of flowing erythrocytes, the SDF device provides pulsed illumination in synchrony with the camera frame rate. This stroboscopic imaging, (partially) prevents smearing of flowing erythrocytes and motion-induced blurring of capillaries due to the short illumination intervals [13].

**Figure 1:**
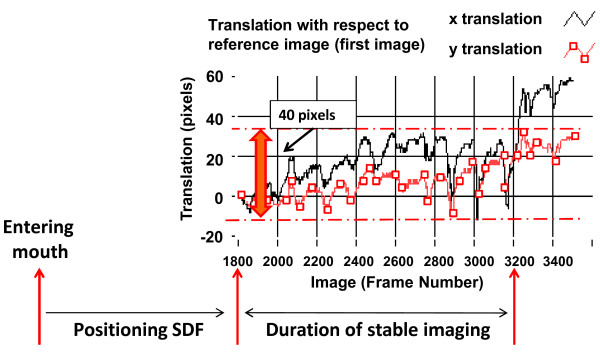
**Assessment of image stability**. Image drifting was measured as the translation (in pixels) of an image with respect to the first image of a video sequence. Image drift of 40 pixels, in either x- or y- direction, was arbitrarily chosen as a cut off for a stable video sequence.

The obtained microcirculatory images (one per time point) were stored on DVI tape and saved onto a computer in DV-AVI file format. The microvessels in the SDF images were analyzed blinded for microvascular diameters and blood flow velocity using a computer software package, MAS (Microvascular Analysis Software, Microvision Medical BV) [14]. Furthermore, image drifting was measured as the translation (in pixels) of an image with respect to the first image of a video sequence as depicted in fig. 1. Image drift of 40 pixels, in either x- or y-direction, was arbitrarily chosen as a cut off for a stable video sequence.

### Image Acquisition Stabilizer (IAS)

**Figure 2:**
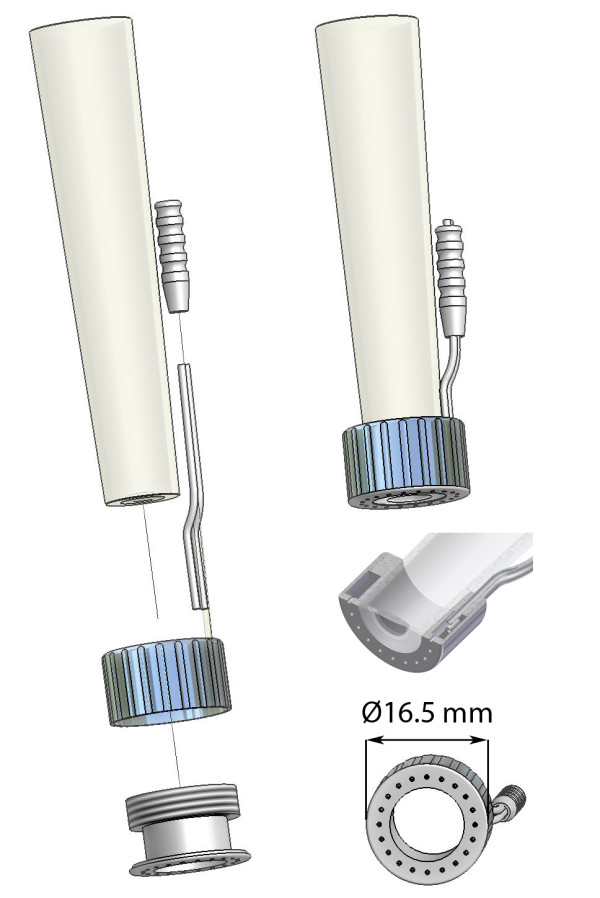
**Image Acquisition Stabilizer**. The sterile Sidestream Dark Field (SDF) imaging cover with the image acquisition stabilizer (IAS) in exploded view (left), attached view (upper right), crosssectional view (middle right), and bottom view (lower right).

The basis of the IAS is a hollow stainless steel cylinder which fits snugly around the tip of the disposable sterile cap of the SDF probe (Fig. 2). The outer cylinder of the IAS can be unscrewed from the inner cylinder to allow cleaning and sterilization of the IAS. Furthermore, the IAS is designed such that it leaves a 100 μm space between the tip of the SDF probe cover and the tissue and thereby relieves the imaged tissue area from pressure without losing image focus. Negative pressure can be applied by use of a readily available bed side vacuum source and a pressure regulator (Digital Vacuum Regulator, Amvex, Richmond Hill, Canada) and applied to the tissue via 20 concave channels. To prevent fluid from reaching the vacuum regulator, a fluid trap (Argyle Lukens Specimen Container; Kendall/Tyco Healthcare/Covidien; Wollerau; Switzerland) is interposed between the IAS and the vacuum regulator. In accordance to previous published values [12], we found the best fit for efficacy of image stabilization and subject's tolerance to lie within 100 ± 20 mmHg of negative pressure (data not shown). The design of the IAS was conceived in collaboration with the Sterilization Department of the Academic Medical Center to adhere to clinical standards for a device which could be re-used. The main requirement for adequate cleaning and sterilization was the possibility to disassemble the device which represents a major difference to the design of Lindert *et al. *[12]. This requirement was implemented in the design as can be seen in figure 2.

### Validation protocol

For the validation part of the study, five experienced SDF imaging operators (i.e., >1 year of SDF imaging training in supervision of the Department of Translational Physiology, Academic Medical Center, Amsterdam) measured sublingual microvascular perfusion in five healthy awake volunteers (n = 5). In case of secretions, the recording was stopped and the awake subjects were asked to swallow. Then, the probe was repositioned and recording was started again. No wipes or cotton sticks were used to absorb saliva as this can cause microscopic bleeding and significantly alter the measurements.

First, the effects of applying peripheral negative pressure on the microcirculatory blood flow velocities [μm/s] were evaluated by switching the negative pressure source on and off during a single video sequence while the SDF device was hand-held. Second, the SDF imaging device was mounted in a force-measuring mechanical arm (Pesola AG, Baar, Switzerland) and the force [N] required to induce pressure artifacts (i.e., stopped or slowed venular flow) was determined with and without the IAS by systematically increasing the force applied by the SDF probe onto the sublingual tissue. Third, the time [s] required for obtaining a stable image sequence and, fourth, the duration [s] of maintaining that stable image sequence were measured.

### Clinical protocol

To demonstrate the clinical applicability of the SDF setup with the IAS, simultaneous bilateral sublingual SDF measurements were conducted in eight intensive care patients undergoing a standard lung recruitment maneuver with one hand-held SDF device and one SDF device mounted in a mechanical arm and equipped with the IAS. This procedure, with a stepwise increment of tidal volume, was chosen in order to create extensive movement artifacts in the sublingual imaged areas. When both SDF devices were acquiring stable images, without pressure artifacts, continuous recording of the video image was started. The lung recruitment maneuver was performed by increasing the inspiratory pressure level to a target of 40 cmH_2_O, followed by gradually reducing the pressure until the baseline ventilator settings were regained. The fraction of inspired oxygen and positive end-expiratory pressure were maintained at 40% and 12 cmH_2_O, respectively, throughout the procedure.

SDF images were recorded non-stop from 1 min before till 1 min after the recruitment maneuver and the recorded SDF video sequences were randomized and analyzed off-line for lateral image drift [μm], drifting velocity [μm/s], and number of loss of image stability (i.e., image drift of > 40 pixels, Fig. 1), the duration [s] that a stable image could be maintained.

### Statistical analysis

Statistical analysis was performed using GraphPad Prism version 5.0 for Windows (GraphPad Software, San Diego, CA, USA). To test data sets for (non-)parametric distributions a D'Agostino-Pearson omnibus normality test was applied. Comparative analysis between data sets was performed with the unpaired Student's t-test or the Mann-Whitney U test and comparative analysis between time points was performed with the paired Student's t-test or the Wilcoxon signed rank test, as appropriate. Differences with a p-value of < 0.05 were considered statistically significant. Results are reported as mean ± SEM.

## Results

### Validation protocol

**Figure 3:**
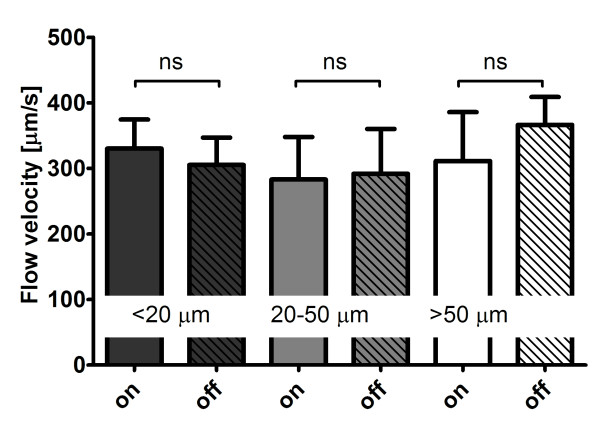
**Influence on microcirculatory flow velocities**. The flow velocity [μm/s] in small (< 20 μm), medium (20-50 μm), and large (> 50 μm) microvessels measured in Sidestream Dark Field (SDF) video sequences of 5 healthy volunteers while the negative pressure source of the image acquisition stabilizer (IAS) was switched on and off. In all microvessels p = ns for negative pressure source on versus off.

The application of peripheral negative pressure thought the IAS did not affect the blood flow velocity in small (< 20 μm), medium (20-50 μm), and large (> 50 μm) microvessels (Fig. 3). The force applied by the SDF imaging probe onto the mucosal tissue required to induce pressure artifacts (i.e., stopped or slowed venular flow) was significantly (p < 0.001) higher with the IAS (0.62 ± 0.05 N) compared to without the IAS (0.25 ± 0.15 N). The time required to obtain a stable SDF image sequence was similar (p = 0.12) with (99 ± 20 s) and without the IAS (150 ± 25 s). The duration of maintaining that stable image was approximately five times longer with the IAS: 8 ± 2 s without the IAS and 42 ± 8 s with the IAS (p < 0.001).

### Clinical protocol

In the eight patients undergoing a lung recruitment maneuver (four male and four female), aged 66 ± 5 years, the APACHE II, APACHE IV, SOFA scores were 19 ± 2, 75 ± 10, and 8 ± 1 points respectively. Four patients were diagnosed with abdominal sepsis, one with coma after cardiac arrest, two had undergone cardiovascular surgery, and one head and neck surgery.

During the lung recruitment maneuver, inspiratory pressure level was increased from 11.4 ± 0.8 cmH_2_O to 43 ± 2 cmH_2_O (p < 0.001). Tidal volume rose accordingly from 423 ± 23 ml (baseline) to 1208 ± 90 ml (p < 0.001) during the lung recruitment maneuver and returned to 477 ± 30 ml after the maneuver (p = 0.017 vs. baseline).

Continuous recording of the SDF video image was started prior to the lung recruitment maneuver when both SDF devices were acquiring stable images, without pressure artifacts. During the procedure, image drift in x- and y-direction was 8.9 ± 2.6 and 10.1 ± 2.0 mm respectively without the IAS, while the drift was reduced to 3.4 ± 0.9 (p = 0.066) and 3.8 ± 1.3 mm (p = 0.018) with the IAS. Drift velocity in x- and y-direction was 15.5 ± 3.9 and 18.4 ± 3.3 μm/s respectively without the IAS, which was reduced to 5.4 ± 1.5 (p = 0.032) and 5.6 ± 1.7 μm/s (p = 0.004) with the IAS. Image drift of > 20 pixels within one video sequence occurred 50 ± 13 times without the IAS and 8 ± 3 times (p < 0.001) with the IAS. The maximum duration of stable imaging during the lung recruitment maneuver was 24 ± 5 s without the IAS and 66 ± 14 s (p = 0.006) with the IAS.

## Discussion

In the present study we developed, evaluated, and validated an IAS for the SDF device. The IAS was based on creating adherence of the SDF probe to the sublingual tissue by applying negative pressure to the periphery of the microscopic field of view. The main findings were that: 1) the IAS did not affect microcirculatory perfusion in the SDF imaging field of view; 2) the IAS prevented pressure artifacts up to a significantly greater force applied by the SDF probe onto the tissue; 3) the time required to obtain a stable image sequence was similar with and without the IAS; and 4) the duration of maintaining that stable image sequence was significantly increased with the IAS. Ultimately, to demonstrate the clinical applicability of the SDF setup with the IAS, simultaneous bilateral sublingual SDF measurements were conducted in intensive care patients undergoing a standard lung recruitment maneuver with one handheld SDF device and one SDF device mounted in a mechanical arm and equipped with the IAS. It was shown that the IAS significantly reduced image drifting and enabled the acquisition of significantly longer image sequences. A final and important finding is also that we showed, in proof of concept, that with the IAS it is possible to perform a measurement without the need for an operator by mounting the device on a mechanical arm, leaving the operator free to perform a clinical maneuver.

The design of the IAS presented here is based on an IAS developed by Lindert et al. for OPS imaging, including the negative pressure level of ≈100 mmHg [12]. To show that application of peripheral negative pressure did not affect microcirculatory perfusion in the SDF imaging field of view Lindert et al. measured blood flow velocities in venules and arterioles. They found that the velocities did not change after switching the negative pressure source on. In the present study, for validation purposes, we investigated the effects on blood flow velocities in small, medium, and large microvessels in five healthy volunteers and provided evidence that indeed microcirculatory perfusion is not affected by application of negative pressure though the IAS. These experiments demonstrated that the IAS is a valid method for SDF image stabilization, not affecting microcirculatory perfusion in the microscopic field of view.

It has been well established that pressure artifacts are easily induced and diminish the reliability of SDF measurements of microcirculatory perfusion [6,11]. This appreciation known from daily application of SDF imaging is confirmed and highlighted by the low force level required to induce pressure artifacts found in the present study. The SDF imaging device has a mass of approximately 360 g. The critical force onto the sublingual tissue without the IAS, at which pressure artifacts are induced, was found to amount approximately 1/6 of the mass of the SDF device. Hence, physical feedback is impossible for SDF operators and visual feedback in the microcirculatory images is necessary to avoid excessive pressure. In fact, most SDF operators use visual feedback to gauge the pressure exerted by the SDF probe on the imaged microcirculation as exemplified in a recent publication [6]. De Backer *et al*., defined the critical pressure inducing perfusion artifacts at the point where venular flow either stopped or significantly slowed down [11]. Using a similar cut-off in the present study we were able to show that the larger surface contact area created by the presence of the IAS resulted in an approximately five times greater force required for the induction of pressure artifacts. This significantly improved SDF image acquisition.

Another important advantage of using an IAS for SDF imaging is that it allows acquisition of longer and more stable SDF image sequences. Previous studies reported that SDF measurements have low intra- and inter-observer variability [3] and that microcirculatory density and perfusion vary highly per site and in time [15]. Hence, studying the microcirculation under pathophysiological conditions requires multiple measurements per time point in order to eliminate this site- and time-dependency of the obtained results. The current microcirculatory image acquisition guidelines dictate that microcirculatory density and perfusion should be measured in 3-5 sites per time point to allow adequate interpretation of the results [11]. Furthermore, according to these guidelines, the length of each SDF image sequence should be > 20 s. This was proven to be rather difficult without the IAS and fairly easy with the IAS. An alternative for multiple measurements to determine the microcirculatory state at a certain time point, continuous measurements of microcirculatory perfusion and density during a clinical maneuver or intervention (e.g., nitroglycerin administration) would allow direct assessment of their effects on the microcirculation. The presented IAS would potentially enable such studies.

Non-invasive intravital imaging modalities, such as OPS and SDF imaging, have been used in studies for monitoring the severity of shock and efficacy of resuscitation and alterations in sublingual microcirculatory density and perfusion have been associated with patient morbidity and mortality [3,6,16]. 'Normalizing' microcirculatory density and perfusion has become focus of new clinical studies and microcirculatory images are gaining a more prominent role in clinical monitoring. Adequate interpretation of microcirculatory images is essential and relies heavily on the quality of the images, in terms of axial and lateral stability. In the present study we showed that the IAS improves both axial and lateral stability of the acquired microcirculatory images and significantly reduced pressure artifacts and image drifting.

## Conclusions

The present study has validated the use of an IAS for improvement of SDF imaging by demonstrating that the application of peripheral negative pressure though the IAS does not affect microcirculatory perfusion in the microscopic field of view. Furthermore, the IAS was shown to improve both axial and lateral SDF image stability and thereby increased the critical force required to induce microcirculatory pressure artifacts and increased the duration of stable image acquisition.

## Key Messages

• The application of peripheral negative pressure though the image acquisition stabilizer (IAS) for improvement of SDF imaging did not affect microcirculatory perfusion in the microscopic field of view.

• The IAS improved both axial and lateral SDF image stability and thereby increased the critical force required to induce microcirculatory pressure artifacts.

• The IAS increased the duration of stable image acquisition.

## Abbreviations

IAS: Image acquisition stabilizer; SDF: Sidestream Dark Field; PA: pressure artifacts; LRM: lung recruitment maneuver; OPS: Orthogonal Polarization Spectral; APACHE score: Acute Physiology And Chronic Health Evaluation score; SOFA score: Sequential Organ Failure Assessment score; DVI: Digital Visual Interface; DV-AVI: Digital Video-Audio Video Interleave.

## Competing interests

CI is inventor of SDF imaging in an Academic Medical Center owned patent and holds shares in Microvision Medical. Furthermore, CI has received research grants from Hutchinson Technology, Baxter, Eli Lilly, and Novartis. The remaining authors declare no potential conflicts of interest.

## Authors' contributions

GMB designed and performed the validation and clinical measurements, analyzed the data of the validation protocol, and drafted the manuscript. RB developed the IAS, co-designed the validation protocol, and co-drafted the manuscript. ECB co-performed the clinical protocol. ZYY and AEE performed the validation protocol. MK analyzed the data of the clinical protocol. CI conceived the study and reviewed the manuscript. All authors read and approved the final version of the manuscript.

## Pre-publication history

The pre-publication history for this paper can be accessed here:

http://www.biomedcentral.com/1471-2342/10/15/prepub
